# The arbuscular mycorrhizal status has an impact on the transcriptome profile and amino acid composition of tomato fruit

**DOI:** 10.1186/1471-2229-12-44

**Published:** 2012-03-27

**Authors:** Alessandra Salvioli, Inès Zouari, Michel Chalot, Paola Bonfante

**Affiliations:** 1 Dipartimento di Biologia Vegetale, Università degli Studi di Torino and IPP-CNR, viale Mattioli 25, 10125 Torino, Italy; 2 IPP-CNR, viale Mattioli 25, 10125 Torino, Italy; 3 Université Henri Poincaré - Nancy I, Faculté des Sciences et Techniques, UMR INRA/UHP 1136 Interactions Arbres/Micro-organismes, BP 239, 54506, Vandoeuvre-les Nancy Cedex, France

## Abstract

**Background:**

Arbuscular mycorrhizal (AM) symbiosis is the most widespread association between plant roots and fungi in natural and agricultural ecosystems. This work investigated the influence of mycorrhization on the economically relevant part of the tomato plant, by analyzing its impact on the physiology of the fruit. To this aim, a combination of phenological observations, transcriptomics (Microarrays and qRT-PCR) and biochemical analyses was used to unravel the changes that occur on fruits from Micro-Tom tomato plants colonized by the AM fungus *Glomus mosseae*.

**Results:**

Mycorrhization accelerated the flowering and fruit development and increased the fruit yield. Eleven transcripts were differentially regulated in the fruit upon mycorrhization, and the mycorrhiza-responsive genes resulted to be involved in nitrogen and carbohydrate metabolism as well as in regulation and signal transduction. Mycorrhization has increased the amino acid abundance in the fruit from mycorrhizal plants, with glutamine and asparagine being the most responsive amino acids.

**Conclusions:**

The obtained results offer novel data on the systemic changes that are induced by the establishment of AM symbiosis in the plant, and confirm the work hypothesis that AM fungi may extend their influence from the root to the fruit.

## Background

Arbuscular mycorrhizal (AM) symbiosis is a widespread mutualistic association, which involves most land plants, including agriculturally relevant species, and plays an important ecological role mainly in the functioning of low-input environments [1]. The microbial partners of this symbiosis are AM fungi, which belong to the Glomeromycota phylum and have particular biological features, being multinucleated obligate biotrophs [1]. As a result of a complex molecular dialogue with their host plants [2], they colonize the plant root cortex and develop intercellular hyphae and highly branched structures called arbuscules within the cells.

The success in time and space of AM symbiosis is mostly due to the benefits that both partners gain, which are above all due to a reciprocal nutrient exchange. The fungal partner plays a key role in providing its host plant with nutrients, mainly phosphorus and nitrogen, which are taken up from the bulk soil by its mycelium and transferred through the symbiotic interface to the plant root cells [3,4]. In turn, the plant supplies the fungus with about 10-20 percent of its net photosynthates [5]. Besides the plant growth stimulation effect, due to the enhanced mineral nutrition, host plants gain multiple benefits from AM symbiosis, i.e. protection from pathogens [6], tolerance to water stress [7,8] and pollutants[9,10], and take advantage of an improved soil structure [11,12].

As a consequence, AM fungi are currently considered key players in agronomic practices as they may lead to a reduction in the use of chemical fertilizers and pesticides, and are therefore potentially important components for the sustainable management of agricultural ecosystems [13].

The exploitation of an AM association, on one hand, requires the development of efficient and controlled fungal inocula, and on the other a careful understanding of the plant physiology upon fungal colonization. To this aim, many high-throughput transcriptomic analyses have been performed on mycorrhizal roots in order to identify mycorrhiza-responsive plant genes. Using a macroarray technique, Liu and colleagues [14] first demonstrated that the colonization of the model plant *Medicago truncatula *by an AM fungus was accompanied by the specific regulation of a number of genes. Later, with the availability of microarray chips, transcriptomic studies were repeated on *M. truncatula *and extended to other plant species [15-22]. Not surprisingly, many regulated plant genes belong to central metabolism, defence mechanisms, and nutrient transport.

Transcriptomic analyses have recently been extended from the target organ (the root) to the whole organism, in order to evaluate whether the long described 'growth effect' observed in AM plants depends on systemic consequences of the association, and whether such an influence entails an organism-wide transcriptional regulation. In 2003, Taylor and Harrier [23] demonstrated that mycorrhizal tomato plants show differential gene expression in roots and also in leaves. Later, García-Rodríguez *et al*. [24] reported the up-regulation of a gene encoding a putative sugar transporter in the leaves of tomato plants colonized by AM fungi. Liu *et al*. [25] were the first to study the global expression pattern in mycorrhizal plant parts, other than the roots, applying the microarray technology, and proved that a systemic regulation of genes involved in stress defence mechanisms is induced in shoots by mycorrhizal fungi. Genes that were differentially regulated were involved in primary and secondary metabolisms, defence and response to stimuli, cell organization, protein modification and transcriptional regulation.

All these data support the hypothesis that, upon colonization, plants activate an organism-wide reprogramming of their main regulatory networks and show that mobile factors of fungal or plant origin are involved in a generalized metabolic change [4].

In addition to legume model plants, many studies on AM transcriptomic changes have been performed using tomato as a host plant [17,19,20,22]. *Solanum_lycopersicum *is an important plant for human nutrition, especially for the so-called low-fat Mediterranean diet, and thanks to the availability of genome sequence data (http://solgenomics.net/genomes/Solanum_lycopersicum/index.pl) and mutant collections (http://www.kdcomm.net/~tomato/Tomato/mutant.htmlhttp://www.agrobios.it/tilling/index.html), it has become one of the agronomically-relevant model plants in mycorrhiza research. In spite of the high number of investigations, limited attention has so far been paid to the influence of AM formation on the physiology of the fruit, the economically relevant part of the tomato plant [26-28]. To date, only one study investigated this question from a molecular point of view, by evaluating the response to mycorrhization of genes encoding for putative allergens in tomato fruit [29]. In this context, our study was aimed at elucidating whether AM mycorrhization has an impact on the fruit metabolism of *Solanum lycopersicum*. We selected the AM fungus *Glomus mosseae *and the Micro-Tom tomato cultivar. Micro-Tom is a dwarf cultivar of tomato, which is characterized by the presence of several mutations, including the dwarf [d] and self-pruning [sp] alleles responsible for its reduced size [30], as well as the resistance to two pathogenic fungi [31]. Based on its small size and short life cycle, the Micro-Tom cultivar has becoming a model system for laboratory purposes, being largely employed for researches on tomato [32,33] with a particular focus on fruit development [34-37]; The features of the Micro-Tom cultivar well fit our study which requires to monitor the plant along its whole life cycle. We in fact followed the plant development till the fruit formation, and focused our attention on the fruit transcriptome by performing a microarray experiment to determine the gene expression profiles in the fruits from mycorrhizal plants. Our transcriptomic analysis was based on the TOM2 oligo-array, which contains about 12000 unigenes, and which is estimated to include about half the genes expressed in fruit [38]. We demonstrate that only a limited number of genes were differentially regulated in the fruit from mycorrhizal plants, most of which were involved in ripening and N metabolism. Then, since in tomato free amino acids increase dramatically during fruit ripening, influencing both the nutritional value and flavour [39,40], we performed a biochemical analysis to elucidate whether mycorrhization has an impact on the amino acid content of tomato fruit, following its quantitative and qualitative evolution throughout the ripening process. The obtained results conclusively show that AM fungi influence the amino acid content of tomato fruits, thanks to an either direct or indirect mechanism.

## Results

### The impact of mycorrhizal inoculation on the growth, phenology and fruit production of tomato plants

We investigated the impact of the AM fungus *Glomus mosseae *on the growth, phenology and fruit production of tomato plants by growing them in pots under controlled conditions. No significant differences were noticed for the plant height, with a mean value of 11.8 cm for the control and 12.46 cm for the myc plants; the inoculated plants (myc) displayed a colonization frequency (F) of 73% and a mycorrhizal intensity (M) of 48.4% in the root system (Additional file 1).

**Figure 1:**
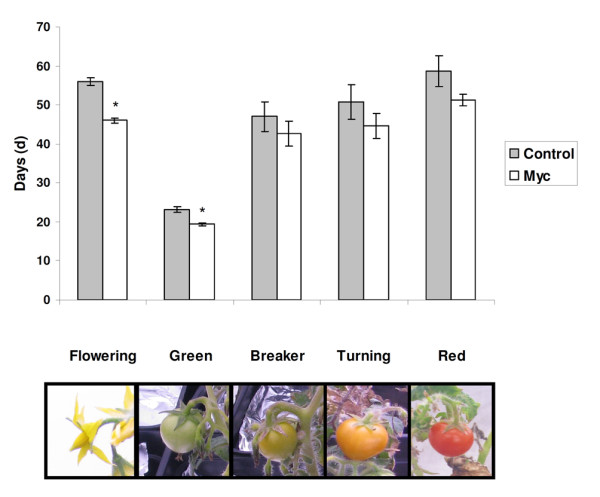
**Flowering time and number of days necessary for the fruit to reach the breaker, turning and red ripening stages**. The flowering time was measured from transplanting date, while for all the other time points, the columns represent the number of days after flowering. Data are presented as the mean ± SE for n = 5. One-way ANOVA (Tukey's posthoc test) was used for data analysis and asterisks indicate statistical significance at p < 0.05.

Flowering and fruit production were also monitored in the myc and control plants to test the effect of AM inoculation on the reproduction processes. Only the development of the first flower/fruit was considered for all the plants, to ensure measurement homogeneity. The mean flowering date (defined as the date at which 50% of the plants had produced their first flower) was significantly earlier in the myc condition and occurred at 47 days after transplanting, while the control plants flowered on average 5 days later (Figure 1). Green fruit formation took significantly less time for the myc plants (19 days) than for the control plants (23 days) (Figure 1). For the measurements on fruit ripening, the differences were not statistically significant but the same trend was noted. Indeed, the time needed to reach respectively the breaker and the turning stages was 4 and 6 days shorter for the myc. Similarly, the transition from turning to red stage was 7 days shorter for the myc plants (Figure 1).

Mycorrhization had a positive and significant effect on the number of fruits produced/plant, which was 5.8 in myc plants versus 2.2 in control (Additional file 2), leading consequently to a higher total fruit number in the myc condition (87 and 33 fruits, respectively). In agreement with previous results [41,42], no significant differences were noted for the individual fruit weight (Additional file 2). Nevertheless, the total fruit yield was higher in mycorrhizal (128.76 g) than in control conditions (61.05 g). The plant shelf-life was also positively influenced by mycorrhization. The control plants showed a progressive loss of vigour and half of them died approximately six months after transplanting, whereas the myc plants continued to produce fruit for at least six more months (data not shown).

### Transcriptomic analysis

In order to investigate the effect of AM inoculation on the global transcriptomic profile of the tomato fruit, a microarray analysis was performed by comparing the RNA from fruit harvested at the turning as defined in Gillaspy *et al*. [43]. Three biological replicates were considered, each made of a pool of RNAs extracted from four different fruits. The RNA was used to hybridize the TOM2 microarray which contains approximately 12,000 tomato unigenes.

The analysis of the microarray results revealed that twenty genes showed a differential expression in the myc condition when compared to the control condition (Additional file 3), seven of them being up-regulated and thirteen down-regulated. When this reduced set of differentially-expressed genes was compared with those already identified in the roots of the myc and control plants, a set of common genes was established.

The up-regulation of the acidic ribosomal protein P1a-like (SGN-U213801) was consistent with data from Hohnjec *et al*. [15], who found evidence of the AM-induced expression of different ribosomal proteins in *Medicago truncatula *roots. With respect to the hormone response, up-regulation of the auxin and ethylene responsive GH3-like protein (SGN-U225740) has already been reported in AM induced genes in tomato [17] and *Medicago truncatula *[15] roots. A Ser/Thr protein kinase (SGN-U221695) was found to be repressed in our experiment, while Hohnjec *et al*. [15] reported that a gene coding for the same function was myc-induced. Interestingly, we did not detect any common gene among those reported as being differentially expressed in the shoots of the mycorrhizal plants [17].

QRT-PCR experiments were performed on the transcripts found to be differentially expressed to confirm the microarray expression results. This experiment was performed twice, with the same RNA used for the microarray analysis and with another set of RNAs independently extracted from the myc and control plants. The results confirmed the expression trend of the twenty tested genes, with the exception of the mads-box protein TAGL12 transcription factor. However, only eleven transcripts were statistically validated (p < 0.05), four out of them being up regulated and seven down regulated. The fold change values ranged from 1.62 to 5.85 for the up-regulated and from 0.6 to 0.18 for the down-regulated genes. Linear regression analysis of the qRT-PCR and microarray data of these genes gave a slope of 1.078 and a value of R^2 ^= 0.876, suggesting a strong correlation between the results of the two approaches (Additional file 4).

### Annotation and putative function of differentially regulated genes

The annotation of the eleven validated genes (Table 1) revealed putative functions that are broadly related to nitrogen, carbohydrate and secondary metabolisms as well as signal transduction pathways.

**Table 1:** Fold changes and putative functions of the differentially regulated genes revealed by microarrays and validated by qRT-PCR

Putative annotation	Fold change (microarray)	Fold change (qRT-PCR)	Molecular Function	Biological Process
histidine decarboxylase *Solanum lycopersicum*	6,75	5,85	Histidine decarboxylase activity pyridoxal phosphate binding	Carboxylic acid metabolic process

acidic ribosomal protein P1a-lik*e Solanum tuberosum*	3,84	1,62	Structural constituent of ribosome	Translational elongation

allantoinase *Vitis vinifera*	2,01	2,76	Allantoinase activity	Allantoin assimilation pathway

alcohol acyl transferase *Solanum lycopersicum*	1,99	3,02	Transferase activity, transferring acyl groups other than amino-acyl groups	Ester biosynthesis

glycolate oxidase *Hyacinthus orientalis*	0,38	0,18	(S)-2-hydroxy-acid oxidase activity	Photorespiration

tcp family transcription factor *Arabidopsis thaliana*	0,4	0,49	Transcription factor activity	Regulation of transcription

glycerophosphoryl diester phosphodiesterase *Arabidopsis thaliana*	0,49	0,33	Glycerophosphodiester phosphodiesterase activity	Glycerol metabolic process Lipid metabolic process

putative protein kinase *Oryza sativa*	0,49	0,41	Protein kinase activity Nucleotide binding	Protein amino acid phosphorylation

udp-glucose 4-epimerase *Solanum tuberosum*	0,56	0,56	UDP-glucose 4-epimerase activity; Coenzyme binding	Galactose metabolic process

Serine/threonine protein kinase A. *thaliana*	0,61	0,6	Protein binding Protein serine/threonine kinase activity	Protein amino acid phosphorylation

galactose-1-phosphate uridylyltransferase *Vitis vinifera*	0,54	0,39	Zinc ion binding UDP-glucose:hexose-1-phosphate uridylyltransferase activity	Galactose metabolic process

Three biological replicates were considered for each experiment. Data were analyzed by one-way ANOVA with Tukey's posthoc test, *p *< 0.05

The gene coding a histidine decarboxylase (SGN-U212615) was the most up-regulated one. This gene is involved in the carboxylic acid metabolic process, since its product is known to catalyze the reaction that produces histamine from histidine. The alcohol acyltransferase gene (SGN-U212578) was also up-regulated. This gene encodes an enzyme that catalyzes the last step in the production of esters, important aroma components in many fruit species [44,45]. An increase in the transcripts encoding for a putative P1a-like ribosomal protein (SGN-U213801) was recorded in the fruit from the myc plants. The gene product plays a crucial role in protein synthesis as it is directly involved in the translation process. The fourth up-regulated gene (SGN-U214730) encodes for a putative allantoinase, which catalyzes the hydrolysis of allantoin to form allantoic acid. Allantoin and allantoic acid are nitrogen-rich organic compounds that play essential roles in the assimilation, metabolism, transport, and storage of nitrogen in plants [46].

Seven genes were confirmed as down-regulated by qRT-PCR. The strongest down-regulated gene was glycolate oxidase 2 (SGN-U232243). This fruit-specific isoform appears to be involved in the photorespiratory pathway [47]. Some of the down-regulated genes encode for proteins involved in the biosynthesis of UDP-sugars, important substrates for the synthesis of plant cell-wall components such as cellulose. This is the case of galactose-1-phosphate-uridylyltransferase (SGN-U218394), and the UDP-glucose-4-epimerase (SGN-U217519) [48]. Again, a third down-regulated gene encodes for the glycerophosphoryl diester phosphodiesterase enzyme (SGN-U216735), which plays an important role in the glycerophospholipid metabolism and has already been detected in the cell wall of carrot [49] and *Arabidopsis thaliana *[50].

Two genes reported as down-regulated encode for proteins with kinase activity; such a function is known to play a key role in eukaryotic cell signalling, and may be involved in the regulation of fruit development [51].

The last down-regulated transcript encodes for the TCP transcription factor (SGN-U223075). This protein family is involved in the regulation of transcription and in the response to gibberellin and abcissic acid stimuli, being involved in the control of lateral shoot morphology in *A. thaliana *[52] and of meristem activation processes in *Solanum tuberosum *[53].

### Analysis of the amino acid content

Considering the up-regulation of the transcripts involved in N assimilation and metabolism, together with the well acknowledged role played by AM fungi in the N metabolism [54], we reasoned that amino acids could be good candidates to test the hypothesis that the mycorrhizal status may have an impact on fruit metabolism.

The free amino acid content was determined in the fruit of the myc and control tomato plants, considering three ripening stages: mature green, turning and red ripe.

The evolution of amino acid content during the ripening is summarized in the additional file 5. During this process, the fruit amino acid content increased significantly in both the control and the myc plants. This is in agreement with other previous data showing that the total fruit amino acid content increases during the ripening process [40]. When the only transition from green to turning stage is considered, the total free amino acid content remained almost unchanged in the fruit of the control plants, whereas it increased by 68% in the fruit from the myc plants. This difference is mainly due to important increases in the asparagine, glutamine and glutamate contents in the fruit of the myc plants, which were more moderate in the fruit of control plants. The increase of the total amino acid content in the red fruit compared to the green was mostly due to significant increases in aspartate by 34- and 41-fold in the control and myc plants, respectively. Asparagine, glutamine and glutamate also increased during ripening but not significantly.

**Figure 2:**
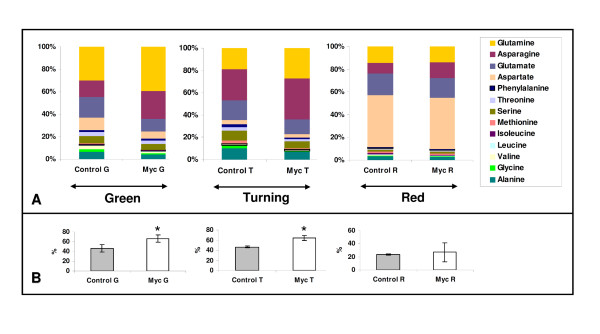
**Aminoacid composition in tomato fruits from control and mycorrhizal (myc) plants**. A. The columns illustrate the relative contribution of the different amino acids to the total pool for the fruit of control and myc plants in three ripening stages: Green (G), Turning (T) and Red (R). B. To evaluate the differences of the two conditions (myc and control plants) for the more abundant amino acids (Asparagine and Glutamine) during the three ripening stages, their values were pooled and subjected to a statistical analysis using one-way ANOVA (Tukey's posthoc test). Asterisk indicates statistically significant differences (*p *< 0.05).

A stage-by-stage comparison of the amino acid content in the fruit from the myc and control plants is summarized in Table 2 and revealed that alanine, serine, threonine and glutamine contents were significantly higher in the fruit from the myc plants in the turning stage. In this ripening stage, the fruit from the myc plants showed a significantly higher free amino acid content than the control plants as the amino acid content in the fruit from the myc plants was 2.5 fold that of the fruit from the control plant. A graphical representation of the amino acid composition in tomato fruits from control and myc plants revealed some differences in the global profile limitedly to the green and turning phase (Figure 2A). The amino acids which mostly contributed to such differences were asparagine and glutamine (Figure 2A), whose contributions - once considered together- reached 43% and 64% in control and myc plants, respectively. These values were confirmed to be significantly different (Figure 2B). This difference appeared to be reduced in the red stage, where the amounts of asparagine and glutamine in the myc fruit returned to be comparable to that of control (Figure 2A).

**Table 2:** Stage- by- stage comparison in amino acid content (nmol/mg dw) between fruit from control and myc plants

	Green		Turning		Red	
	**Control**	**Myc**	**Control**	**Myc**	**Control**	**Myc**

**Alanine**	1,282 ± 0,455	1,176 ± 0,538	1,993 ± 0.200	**3,389 ± 0.099**	5,393 ± 0,380	5,120 ± 1,344
**Glycine**	0,365 ± 0,098	0,285 ± 0,148	0,509 ± 0.015	0,394 ± 0.039	1,246 ± 0,526	1,120 ± 0,133
**Valine**	0,644 ± 0,1	0,426 ± 0,085	0,192 ± 0.068	0,432 ± 0.152	0,469 ± 0,034	0,558 ± 0,068
**Leucine**	0,096 ± 0,028	0,145 ± 0,044	0,105 ± 0.027	0,194 ± 0.003	1,437 ± 0,295	0,764 ± 0,118
**Isoleucine**	0,243 ± 0,057	0,326 ± 0,038	0,102 ± 0.011	0,204 ± 0.039	1,156 ± 0,232	1,237 ± 0,146
**Methionine**	0,074 ± 0,017	0,104 ± 0,113	0,495 ± 0.359	0,472 ± 0.085	1,800 ± 0,215	2,024 ± 0,515
**Serine**	1,263 ± 0,162	1,484 ± 0,817	1,645 ± 0.012	**2,899 ± 0.059**	2,785 ± 0,097	2,473 ± 0,195
**Threonine**	0,682 ± 0,165	0,891 ± 0,16	0,617 ± 0.025	**0,944 ± 0.039**	2,257 ± 0,508	2,447 ± 0,780
**Phenylalanine**	0,336 ± 0,039	0,424 ± 0,112	0,502 ± 0.005	0,675 ± 0.091	2,234 ± 0,619	1,782 ± 0,506
**Aspartate**	2,097 ± 0,639	1,995 ± 0,59	0,831 ± 0.150	1,481 ± 0.182	72,496 ± 18,674	81,855 ± 12,443
**Glutamate**	3,509 ± 1,008	3,156 ± 2,163	3,497 ± 0.117	6,632 ± 1.153	29,611 ± 4,284	31,542 ± 7,272
**Asparagine**	2,869 ± 0,439	7,326 ± 3,084	5,386 ± 0.277	17,960 ± 3.757	14,548 ± 0,932	25,472 ± 11,906
**Glutamine**	5,720 ± 0,359	**11,463 ± 1,304**	3,738 ± 0.081	**13,445 ± 0.989**	23,379 ± 7,211	24,913 ± 9,617
**Total**	19,179 ± 3,361	29,202 ± 8,863	19,612 ± 0.003	**49,120 ± 4.011**	158,810 ± 32,210	181,305 ± 25,610

Three ripening stages were considered: mature green, turning and red. Results are presented as mean of three biological replicates ± SE and were analyzed by one-way ANOVA with Tukey's posthoc test, *p *< 0.05. Statistically significant data are highlighted in bold

## Discussion

### Tomato plants respond to AM fungi in a systemic way with phenological changes

This study reveals that mycorrhization causes systemic effects on tomato fruits from Micro-Tom plants leading to phenological, molecular and metabolic variations. Monitoring the fruit production of the Micro-tom tomato plants along their life cycle has shown that mycorrhization increases plant productivity. The positive impact of AM fungi on tomato plant productivity has already been reported [26,27] and explained as being related to the improved nutritional status, which is caused by the higher efficiency of mycorrhizal plants in taking up soil phosphate [26].

In our experiments, tomato phenology has also been shown to be clearly affected by mycorrhization. We noticed a significant acceleration in the transition from the vegetative phase to flowering in the myc plants. This result is in agreement with the findings of Hildebrandt *et al*. [55] who obtained similar results in tomato plants inoculated with *Glomus intraradices*. Two hypotheses can explain the flowering acceleration. First, the improved nutritional status may force the meristem transition from the vegetative to floral status; as an alternative, the fungus might have - not yet characterized - effects on the expression of one (or more) gene related to the flowering. Flowering time is reported to be under the control of microRNAs, i.e. small RNAs which regulate target transcripts through an RNA cleavage mechanism, as well as transcription factors [56,57]. Gu and colleagues [58] identified a group of micro RNAs in tomato leaves which are exclusively induced by the AM symbiosis, independently of the Pi availability. According to this finding, a direct fungal effect on plant phenology could occur by means of a systemic modulation of the transcription, and without any direct link to the already stated plant growth promotion.

Under our experimental conditions, mycorrhization has also been shown to lead to a significant faster fruit production (Figure 1, Green phase), while not statistically significant differences were reported for the transition from one ripening stage to another. This effect, obtained in two independent experiments, has never been described before, and opens the question on whether mycorrhization may have a direct or indirect effect on the fruit development process. Although mycorrhization appeared to accelerate fruit development, interestingly, the shelf life of the plant was not negatively affected: if the myc plants began the fruit production earlier, their shelf-life and productive phase were also longer, thus positively influencing fruit production. However, due to the genetic background of the Micro-Tom tomato, a crosstalk among mutations, mycorrhization and ripening cannot be excluded.

### From the root to the fruit: the AM fungus has an impact on the fruit transcriptome

A number of data sets previously demonstrated that AM fungi have an important effect on the transcriptome profile of both roots and shoots in tomato [17,25]. By using TOM2 microarrays, we have demonstrated that *Glomus mosseae *also has an impact on the global gene expression of tomato fruit. The results have shown the differential regulation of twenty genes, and out of these, eleven were statistically validated. These data suggest that mycorrhization could directly influence the gene expression of fruit, thus implying the occurrence of a systemic "myc-effect", which was already demonstrated to extend from roots to shoots [17,25] and leaves [23,24]. Schwarz and colleagues [29] indicated that mycorrhization impacts the transcription of genes involved in red fruit allergens, although no phenotypical changes were recorded and mycorrhization rate was low. In our work, we report that eleven genes were significantly affected by mycorrhization. The TOM2 microarrays are reported to contain only half of the tomato genes expressed in the fruit: it is therefore possible that the actual number of AM-regulated genes in fruit is higher than the one reported here. However, the limited number of regulated genes could mirror the specificity of the metabolic pathways which are regulated by AM fungi in tomato fruit. A limited number of regulated transcripts (fourteen) was also reported by Waller *et al*. [38] in a work on the expression of folate biosynthesis genes during tomato fruit development, demonstrating how tomato fruit pathways are finely regulated.

Interestingly, some (auxin and ethylene responsive GH3 like protein, ribosomal protein encoding gene) of the twenty genes described in our study were also reported to be differentially regulated in tomato roots [17], suggesting that there is a core set of genes which is highly sensitive to (or targeted by) mycorrhization, irrespectively of the organ considered.

A further finding of our research is that some genes known to be related to the ripening process were also regulated by mycorrhization. Such genes are involved in novel protein synthesis, changes in cell wall texture, as well as accumulation of specific compounds. The gene coding for an alcohol acyltransferase (*Aat*) resulted to be up-regulated in the fruit of mycorrhizal plants. The AAT enzyme is involved in the metabolism of volatile esters, major aroma components in many fruit species like apple, melon and strawberry [44,59,60]. Most of the fruit volatile esters are dependent on ethylene, and the *Aat *gene expression was shown to be under the control of such hormone [44,61]. Another up-regulated sequence showed the highest similarity with the histidine decarboxylase gene (*Hdc*) which is up-regulated in the early ripening stages [62]. Decarboxylation of histidine results in the formation of histamine, which is known to be produced in the ripe tomatoes. These results suggest that mycorrhization might directly interact with the fruit ripening process, possibly influencing features related to fruit quality.

The putative ribosomal protein P1a-like encoding gene was found to be up-regulated in our experiments. This group of genes was reported by Carbone *et al*. [63] to be the largest group of up-regulated genes in ripening tomato fruit. This gene family is probably involved in the high protein turnover existing in the ripening fruit.

Mycorrhization had an effect on genes involved in cell wall biosynthesis, too. First, mycorrhization led to the down-regulation of two genes involved in UDP-sugar synthesis: the galactose-1-phosphate uridylyltransferase and UDP-glucose 4-epimerase. The down-regulation of this latter gene leads to a net loss of galactosyl residues in the cell wall [48], an event well described during the tomato fruit ripening [64]. Second, the glycerophosphoryl diester phosphodiesterase (*Gpd*) gene, whose product plays a role in plant cell wall remodelling [50,65] was down-regulated in myc fruit. The increased phosphate availability which accompanies the mycorrhizal status [66] could explain the *Gpd *gene down-regulation. Both the gene and its product were indeed reported to be up-regulated by P deprivation in the roots of diverse plant species [49,67].

These data suggest that mycorrhizal establishment influences gene transcription in tomato fruit, assuming that one systemic factor (or more) could be responsible for such an effect. In the last years, phytohormones such as ethylene, salicylic acid, abscisic acid and jasmonates have been shown to play a crucial role in the establishment and functionality of the AM symbiosis [20,68-70]; they can thus be considered good candidates to explain the systemic effect of mycorrhization on tomato fruit. However, as already stated for the plant growth effect, an indirect effect of AMs on the fruit metabolism *via *the well-known positive effect of symbiosis on nutrient availability to the plant cannot be ruled out.

### Regulation of N-related genes and changes in the amino acid content

Together with phosphate, nitrogen is the key element taken up by AM fungi, and genes involved in the transport of nitrate, ammonium and amino acids have already been identified [54]. The model proposed by Govindarajulu and colleagues [71] includes the synthesis of arginine in the extraradical mycelium and its transfer to the intraradical mycelium, where it is released under different N forms to host plants [54]. The transcriptomic analysis of tomato fruit from myc plants has revealed the up-regulation of a putative allantoinase encoding gene, which catalyses the hydrolysis of allantoin to form allantoic acid. These two nitrogen-rich compounds (also known as ureides) play essential roles in the assimilation, metabolism, transport, and storage of nitrogen in plants [46]. Allantoinases have been widely recognized as being involved in ureide export in legume and non-legume plants [72] and allantoinase gene expression was reported to be positively regulated by allantoin [73]. These results open the question whether these N-rich compounds may have a fungal origin.

An important finding of our investigation was coming out from the qualitative and quantitative amino acid analysis: the fruit of the myc plants had higher total free amino acid content than the fruit of the control plants, and this difference was statistically significant in the turning stage. Looking at the composition in amino acids, the difference resulted to be essentially due to a higher content in glutamine and asparagine.

In order to determine whether this difference was due to a higher expression of the genes responsible for their synthesis in the fruit, we performed qRT-PCR on the glutamine synthetase and asparagine synthetase genes. Since no differences were noted for the gene expression between the fruit of the myc and control plants (data not shown), we hypothesized that these two amides were not newly synthesized in the fruit, but that they moved there from the host root where they have been synthesized.

This hypothesis is well supported by Rutzika *et al*. [22]. They showed that glutamine synthetase and asparagine synthetase were significantly more expressed in tomato mycorrhizal roots, which led to a higher synthesis of glutamine and asparagine in the mycorrhizal roots. In addition, these two amino acids are known to be primary nitrogen transport compounds from source to sink organs where they serve as nitrogen reserve in many plants [74].

As an alternative hypothesis, these amino acids could be synthesized by the AM fungus. Interestingly, Gomez and colleagues [75] reported the expression of *G. intraradices *asparagine synthetase and glutamine synthetase gene expression in arbuscules; they, hence, postulated that ornithine released in the intraradical mycelium [71] may be cycled into asparagine and glutamine through the action of asparagine synthetase and glutamine synthetase. This finding coupled with an accumulation of glutamate, aspartate and asparagine in the mycorrhizal roots [76] and the discovery of a plant amino acid permease specifically induced in myc roots [16] could allow to speculate about a fungal origin for these amino acids.

## Conclusions

In conclusion, for the first time a combination of morphological, molecular and biochemical approaches revealed the presence of multi-level changes in tomato fruit from mycorrhizal plants. The establishment of a mycorrhizal symbiosis in the roots was shown not only to modulate the fruit gene expression, *via *a systemic effect, but also to create a network of effects which lead to changes in the phenology of flowering and fruit ripening as well as in the amino acid profile, suggesting the occurrence of deep metabolic modifications. Hormones can represent a major player in this complex scenario of modifications; interestingly, in tomato fruit, besides the major role played by ethylene in controlling the ripening process [77], recent findings reveal that methyl jasmonate also influence the fruit metabolome and especially the aminome [78], suggesting that the mycorrhiza-induced changes we observed in the fruit can be attributed to a hormonal signalling.

Finally, the results described here may give a support to applicative researches which link arbuscular mycorrhization with the commercially-valuable parts of crops in field conditions. This will lead to a systematic better agricultural exploitation of mycorrhizal symbiosis.

## Methods

### Plant material

*Solanum lycopersicum *cv.Micro-Tom tomato seeds were sterilized with a series of washes: 3 min in 70% ethanol, to which 3-4 drops of tween 20 were added, 13 min in a 5% bleach solution and 3 washes of 10 min each in sterile water. The seeds were then placed in a 0.6% agar medium (5 seeds per petri dish). The petri dishes were kept for 5 days in the dark, followed by 4 days in the light. The germinating seedlings were then transferred to pots with sterile quartz sand. For mycorrhization, the fungus *Glomus mosseae *Gerd. & Trappe (BEG 12) was purchased from Biorize (Dijon, France). A mixture of sand (70%) and fungal inoculum (30%) was used. The mycorrhizal and control plants were grown in a growth chamber under a 14 h light (24°C)/10 h dark (20°C) regime, and watered, 125 ml/plant twice a week with water, and once a week with a modified Long-Ashton solution containing a low phosphorus concentration (3.2 μM Na2HPO4·12H2O) [79]. The inoculated and control plants were observed during their development and the traits related to plant growth, fruit production and phenology, such as flowering time [26] and fruit maturation, were measured. For phenologial data, a statistical analysis was conducted using the Statistica 6 software, applying the one-way ANOVA and Tukey test adopting a probability level of p < 0.05. The statistical analysis of fruit production and fruit weight was conducted using the Kruskal-Wallis non-parametric test.

The fruit was collected when it reached the required ripening stage i.e. turning, as depicted in Figure 1. The seeds were discarded and the pericarp was cut into small pieces, put in 2 ml reaction tubes, frozen immediately in liquid nitrogen and stored at -80°C until required. The fruit pericarps were freeze-dried overnight and stored at -80°C.

At the end of the experiment, the plant roots were cut and the fungal colonization was assessed according to the Trouvelot five-class system [80] using MYCOCALC software. Twelve plants were considered for the root mycorrhization intensity assessment and five microscope slides were analyzed per plant, each slide containing 20 root pieces of 1 cm.

### RNA isolation

RNA was extracted using the 'pine tree-method', with modifications according to Guether *et al*. [16]. The total RNA was quantified with a spectrophotometer (NanoDrop, Technologies Inc.) and wavelength ratios of A260 nm/280 nm ~ 2 and A260 nm/A230 nm ≥ 2 were controlled for RNA purity. For microarray experiments RNA integrity was checked with the Experion system (Bio-Rad) and samples with a RIN value less than 8 were discarded. The RNAs were pooled in three biological replicates for microarray and quantitative RT-PCR experiments; each pool contained RNAs from 4 fruits collected at the turning stage from 4 different plants.

### Microarray experiment

The TOM2 microarrays were obtained from the Center for Gene Expression Profiles (CGEP; Cornell University, Ithaca, NY, USA). Each microarray contains 11769 oligonucleotide probes, whose design was based on gene transcript sequences from the Lycopersicon Combined Built # 3 unigene database (http://www.SGN.cornell.edu). Three biological replicates were analysed and a 'dye swap' approach was adopted. Total RNA (500 ng) was used to generate direct fluorescently labelled cRNA using the Low RNA Input Linear Amp Kit (Agilent) according to the manufacturer's instructions. Slides were treated following the prehybridization protocol provided by the manufacturer (http://ted.bti.cornell.edu/cgi-bin/TFGD/array/TOM2_hybridization.cgi). Microarray hybridization was performed using the Gene Expression Hybridization kit (Agilent). Post-hybridization was performed following the manufacturer's instructions with slight modifications as described in Fiorilli *et al*. [17]. The slides were then scanned using an Agilent microarray scanner (G2565BA) at a resolution of 10 μm and laser power set to 90%. The fluorescence data were processed using IMAGENE software (version 5.6; BioDiscovery Inc.; http://www.biodiscovery.com). Normalization and analysis of the microarray data were performed using the LIMMA software package (BIOCONDUCTOR package) [81]. The values of all the spots on the arrays were per spot and per chip intensity dependent (Lowess) normalized. Significant up- or down- regulated genes were filtered for a false discovery rate of < 0.05 and for greater or lower normalized expression ratios than 1.5- or 0.67-fold, respectively. Gene ontology (GO) term annotation was obtained using the BLAST2GO software [82].

### DNA extraction and PCR

The DNA extraction was performed on 100 mg of leaves using the DNA Plant Mini. Kit (Qiagen) according to the manufacturer's instructions. Sequence data of the differentially regulated transcripts were obtained from the Tomato Functional Genomics Database http://ted.bti.cornell.edu/ using the microarray unigene ID.

Specific primers were designed with Primer3 software (http://frodo.wi.mit.edu/primer3/) and were tested on cDNA for amplification before qRT-PCR. PCR assays were carried out in a final volume of 25 μl containing 2.5 μl of 10X buffer, 1 μl of 2.5 mM dNTPs, 0.4 μl of each primer 10 μM, 1 μl of Red Taq polymerase (Sigma), and 1.5 μl of a total DNA diluted 1:10. The PCR cycling programme consisted of: 95°C for 5 min, 40 cycles of 94°C for 45 sec, 65°C for 45 sec and 72°C for 45 sec. The primer names and corresponding sequences are listed in additional file 6.

### Quantitative RT-PCR

The quantitative RT-PCR experiments were carried out on the same RNA used for the microarray analysis and on another RNA samples extracted from an independent set of fruits from myc and control plants.

All the RNA samples were treated with the Turbo DNA-free™ kit (Ambion, Austin, TX, USA) for qRT-PCR analyses according to the manufacturer's instructions. The RNA samples were submitted to a control reverse-transcription PCR to check for the absence of DNA contamination using the One Step RT-PCR kit (Qiagen) and Ubiquitin tomato primers designed by Fiorilli *et al*. [17].

First strand cDNA was synthesized from 700 ng of total RNA with the Superscript II reverse transcriptase kit (invitrogen) following the manufacturers' instructions. At the end of the reaction, the cDNA was diluted to 1:3 for the gene expression analysis.

Quantitative RT-PCR reactions were carried out in a 48-well StepOne™ Real time PCR system instrument (Applied Biosystems), in a final volume of 20 μl, containing 10 μl of 23 iQ SYBR Green Supermix, 4 μl of primers 3 μM, 5 μl of water and 1 μl of cDNA template. The PCR programme consisted of a holding stage (95°C for 10 min) and 40 cycles of 95°C for 15 sec and 60°C for 1 min.

A melting curve (55-95°C with a heating rate of 0.5°C per 10 sec and a continuous fluorescence measurement) was recorded at the end of each run to assess for amplification product specificity. All the reactions were performed with three technical replicates and three biological replicates. A portion of the ubiquitin gene was used as the housekeeping gene for normalization. PCR efficiency was determined from standard curves constructed from serial dilutions of tomato genomic DNA.

The comparative threshold cycle method ΔΔCt was adopted as the analysis method for the relative RNA expression [83]. The Ct values of the target genes imported by the system were normalized to the Ct values of the ubiquitin, considering the equation: ΔCt = Ct target -Ct housekeeping. Before calculating the ΔCt, the technical replicates were checked for their Ct value uniformity and for outliers, which led to the exclusion of any standard deviations above 0.2. The fold change was calculated from equation 2^- ΔΔCt^, *where *ΔΔCt = ΔCt sample - ΔCt control [83]. A statistical analysis was conducted using the Statistica 6 software, applying the one-way ANOVA and Tukey test adopting a probability level of *p *< 0. 05.

### Determination of the free amino acid content

Freeze-dried pieces of fruit were used for the amino acid quantification. We considered three ripening stages: mature green, turning and red mature. Three biological replicates were used for each stage. Amino acid extraction was performed using the method described in Javelle *et al*. [84].

The results were statistically analysed using the Statistica 6 software, applying the one-way ANOVA and Tukey test adopting a probability level of *p *< 0. 05

## Competing interests

The authors declare that they have no competing interests.

## Authors' contributions

AS participated in designing the experiments, carried out Microarray analysis and data validation; contributed to the manuscript writing. IZ participated in the design of some experiments and the validation of Microarray data, carried out phenotypical and biochemical analyses and contributed to the manuscript writing. MC directed the biochemical analyses, and provided support in their interpretation. PB conceived and coordinated the study, and contributed to the manuscript writing. All authors read and approved the final manuscript.

## Supplementary Material

Additional file 1 **Mycorrhization parameters of tomato roots inoculated with the AM fungus *Glomus mosseae***. Mycorrhization parameters were determined at the end of the experiment according to Trouvelot method (1986). F%: frequency of colonization in the root system; M%: intensity of the mycorrhizal colonization in the root system; A%: arbuscule abundance in the root system; and a%: arbuscule abundance in the mycorrhizal root part. Values indicated at the top of each column represent the mean of the corresponding parameter for n = 12 (root systems from 12 plants) and bars represent the standard deviation. For each plant, 100 cm of root were measured. Click here for file

Additional file 2 **Fruit yield of tomato (cv. Micro-Tom) in control and mycorrhizal conditions**. Values are expressed as mean of fifteen plants ± SD. Statistical analysis of the data was performed using the non-parametric Kruskal-Wallis test. Different letters indicate significant differences (p < 0.05). Click here for file

Additional file 3 **Microarray folds changes and putative annotations of the differentially regulated genes in fruit of mycorrhizal plants**. Click here for file

Additional file 4 **Comparison of microarray and qRT-PCR data for the 11 validated genes**. Each symbol represents the mean fold change (log2 transformed). Click here for file

Additional file 5 **The evolution of the amino acid content (nmol/mg dw) with ripening in the fruit from control and myc plants**. Three ripening stages were considered: mature green, turning and red. Results are presented as mean of three biological replicates ± SE. For the statistical tests, the data of turning and red stages were both compared to those of the green stage and were analyzed by one- way ANOVA with Tukey's posthoc test, p < 0.05. Statistically significant data are highlighted in bold. Click here for file

Additional file 6 **qRT-PCR primers list**. Click here for file
