# Supplementary material for: The arbuscular mycorrhizal status has an impact on the transcriptome profile and amino acid composition of tomato fruit
Source: BMC Plant Biol. 2012 Mar 27;12:44. doi: 10.1186/1471-2229-12-44 (PMC3362744; doi:10.1186/1471-2229-12-44)
Supplement: Additional file 1 — Mycorrhization parameters of tomato roots inoculated with the AM fungus Glomus mosseae. Mycorrhization parameters were determined at the end of the experiment according to Trouvelot method (1986). F%: frequency of colonization in the root system; M%: intensity of the mycorrhizal colonization in the root system; A%: arbuscule abundance in the root system; and a%: arbuscule abundance in the mycorrhizal root part. Values indicated at the top of each column represent the mean of the corresponding parameter for n = 12 (root systems from 12 plants) and bars represent the standard deviation. For each plant, 100 cm of root were measured. [file 1471-2229-12-44-S1.DOC]

Additional file 1: Mycorrhization parameters of tomato roots inoculated with the AM fungus *Glomus mosseae.* Mycorrhization parameters were determined at the end of the experiment according to Trouvelot method (1986). **F%: frequency of colonization in the root system; M%: intensity of the mycorrhizal colonization in the root system; A%: arbuscule abundance in the root system; and a%: arbuscule abundance in the mycorrhizal root part. Values indicated at the top of each column represent the mean of the corresponding parameter for** n=12 (root system from 12 plants) and error bars represent the standard deviation**. For each plant, 100 cm of root were measured.**
