# Supplementary material for: The arbuscular mycorrhizal status has an impact on the transcriptome profile and amino acid composition of tomato fruit
Source: BMC Plant Biol. 2012 Mar 27;12:44. doi: 10.1186/1471-2229-12-44 (PMC3362744; doi:10.1186/1471-2229-12-44)
Supplement: Additional file 2 — Fruit yield of tomato (cv. Micro-Tom) in control and mycorrhizal conditions. Values are expressed as mean of fifteen plants ± SD. Statistical analysis of the data was performed using the non-parametric Kruskal-Wallis test. Different letters indicate significant differences (p < 0.05). [file 1471-2229-12-44-S2.DOC]

**Additional file 2**: **Fruit yield of tomato (cv. Micro-Tom) in control and mycorrhizal conditions**

|  | **Control** | **Myc** |
| --- | --- | --- |
| **Average fruit number/plant** | 2,2**±**1,26 a | 5,8**±**1,47 b |
| **Average fruit weight (g)** | 1,85**±**0,54 a | 1,48**±**0,6 a |
| **Total fruit number** | 33 | 87 |
| **Total yield (g)** | 61,05 | 128,76 |

Values are expressed as mean of fifteen plants ±SD. Statistical analysis of the data was performed using the non-parametric Kruskal-Wallis test. Different letters indicate significant differences (p<0.05).
