# Supplementary material for: The arbuscular mycorrhizal status has an impact on the transcriptome profile and amino acid composition of tomato fruit
Source: BMC Plant Biol. 2012 Mar 27;12:44. doi: 10.1186/1471-2229-12-44 (PMC3362744; doi:10.1186/1471-2229-12-44)
Supplement: Additional file 3 — Microarray folds changes and putative annotations of the differentially regulated genes in fruit of mycorrhizal plants. [file 1471-2229-12-44-S3.DOC]

### Additional file 3: Microarray fold changes and putative annotations of the differentially regulated genes in fruit of mycorrhizal plants.

| **SGN-ID** | **Putative annotation** | **Gene**  **Name** | **Array Fold change** |
| --- | --- | --- | --- |
| SGN-U212578 | alcohol acyl transferase *Lycopersicon esculentum* | AAT | 1,99 |
| SGN-U212615 | histidine decarboxylase *Solanum lycopersicum* | HDC | 6,75 |
| SGN-U213801 | acidic ribosomal protein P1a-like *Solanum tuberosum* | P1a | 3,84 |
| SGN-U218950 | actin depolymerizing factor *Vitis vinifera* | ADF | 1,98 |
| SGN-U214730 | allantoinase *Vitis vinifera* | ALN | 2,01 |
| SGN-U225740 | **auxin and ethylene responsive GH3-like protein *Capsicum chinense*** | GH3 | 3,7 |
| SGN-U233519 | TAGL12 transcription factor mads-box protein- *Lycopersicon esculentum* | TAGL12 | 1,7 |
| SGN-U213123 | arginine decarboxylase *Solanum lycopersicum* | ADC | 0.44 |
| SGN-U214453 | **Probable 26S proteasome non-ATPase regulatory subunit 3** *Nicotiana tabacum* | PRS | 0.25 |
| SGN-U216105 | trehalose-6-phosphate synthase *Solanum tuberosum* | TPS | 0.63 |
| SGN-U216735 | glycerophosphoryl diester phosphodiesterase *Arabidopsis thaliana* | GPD | 0.5 |
| SGN-U217519 | udp-glucose 4-epimerase *Solanum tuberosum* | UGE | 0.56 |
| SGN-U218394 | galactose-1-phosphate uridylyltransferase *Vitis vinifera* | GPU | 0.64 |
| SGN-U221249 | ssu72 RNA polymerase II ctd phosphatase homolog *Gallus gallus* | SSU72 | 0.54 |
| SGN-U221514 | putative protein kinase *Oryza sativa* | APK | 0.5 |
| SGN-U221695 | serine/threonine protein kinase *Arabidopsis thaliana* | STK | 0.61 |
| SGN-U223075 | tcp family transcription factor *Arabidopsis thaliana* | TCP | 0.4 |
| SGN-U232245 | glycolate oxidase *Nicotiana tabacum* | GOX | 0.37 |
| SGN-U232243 | glycolate oxidase *Hyacinthus orientalis* | GOX2 | 0.38 |
| X54029 | heat shock protein 70 *Solanum lycopersicum* | HSP70 | 0.33 |
|  |  |  |  |
