# Supplementary material for: The arbuscular mycorrhizal status has an impact on the transcriptome profile and amino acid composition of tomato fruit
Source: BMC Plant Biol. 2012 Mar 27;12:44. doi: 10.1186/1471-2229-12-44 (PMC3362744; doi:10.1186/1471-2229-12-44)
Supplement: Additional file 4 — Comparison of microarray and qRT-PCR data for the 11 validated genes. Each symbol represents the mean fold change (log2 transformed). [file 1471-2229-12-44-S4.DOC]

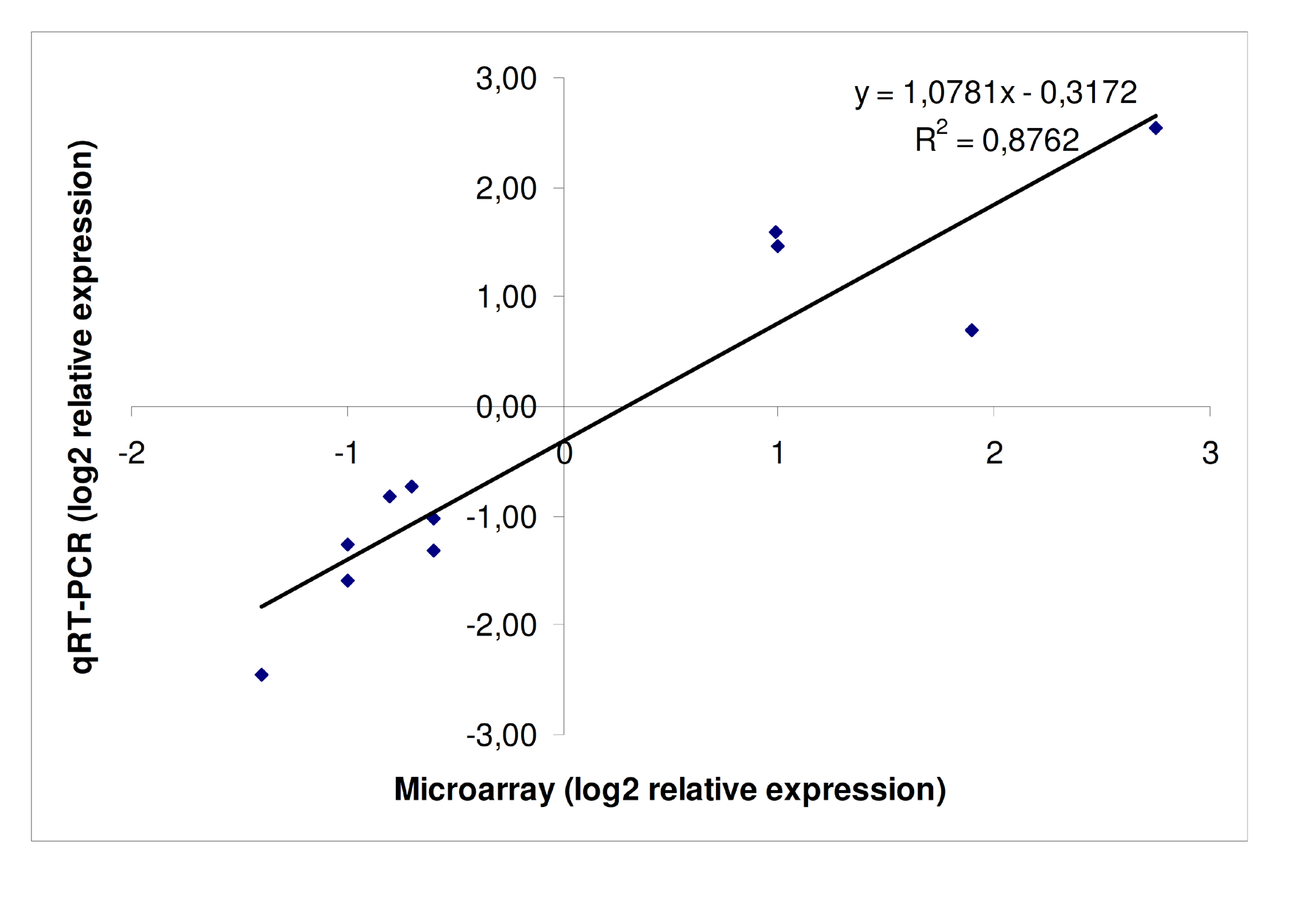
 **Additional file 4: Comparison of microarray and qRT-PCR data for the 11 validated genes listed in table 1**. Each symbol represents the mean fold change (log2-transformed).
