# Supplementary material for: The arbuscular mycorrhizal status has an impact on the transcriptome profile and amino acid composition of tomato fruit
Source: BMC Plant Biol. 2012 Mar 27;12:44. doi: 10.1186/1471-2229-12-44 (PMC3362744; doi:10.1186/1471-2229-12-44)
Supplement: Additional file 5 — The evolution of the amino acid content (nmol/mg dw) with ripening in the fruit from control and myc plants. Three ripening stages were considered: mature green, turning and red. Results are presented as mean of three biological replicates ± SE. For the statistical tests, the data of turning and red stages were both compared to those of the green stage and were analyzed by one- way ANOVA with Tukey's posthoc test, p < 0.05. Statistically significant data are highlighted in bold. [file 1471-2229-12-44-S5.DOC]

**Additional file 5**: **The evolution of the amino acid content (nmol/mg dw) with ripening in the fruit from control and myc plants**. Three ripening stages were considered: mature green, turning and red

|  |  |  | **Control** | |  |  | **Myc** | | | |  | |
| --- | --- | --- | --- | --- | --- | --- | --- | --- | --- | --- | --- | --- |
|  | **Green** | | **Turning** | | **Red** | | **Green** | | **Turning** | | **Red** | |
| **Alanine** | 1.282 **±** | 0.455 | 1.993**±** | 0.200 | 5.393 **±** | 0,380 | 1.176 **±** | 0.538 | **3.389±** | **0.099** | 5.120 **±** | 1,34 |
| **Glycine** | 0.365 **±** | 0.098 | 0.509**±** | 0.015 | 1.246 **±** | 0,526 | 0.285 **±** | 0.148 | 0.394**±** | 0.039 | 1.120 **±** | 0,13 |
| **Valine** | 0.644 **±** | 0.100 | 0.192**±** | 0.068 | 0.469 **±** | 0,034 | 0.426 **±** | 0.085 | 0.432**±** | 0.152 | 0.558 **±** | 0,07 |
| **Leucine** | 0.096 **±** | 0.028 | 0.105**±** | 0.027 | **1.437 ±** | **0,295** | 0.145 **±** | 0.044 | 0.194**±** | 0.003 | **0.764 ±** | **0,12** |
| **Isoleucine** | 0.243 **±** | 0.057 | 0.102**±** | 0.011 | **1.156 ±** | **0,232** | 0.326 **±** | 0.038 | 0.204**±** | 0.039 | **1.237 ±** | **0,15** |
| **Methionine** | 0.074 **±** | 0.017 | 0.495**±** | 0.359 | 1.800 **±** | 0,215 | 0.104 **±** | 0.113 | 0.472**±** | 0.085 | 2.024 **±** | 0,51 |
| **Serine** | 1.263 **±** | 0.162 | 1.645**±** | 0.012 | 2.785 **±** | 0,097 | 1.484 **±** | 0.817 | 2.899**±** | 0.059 | 2.473 **±** | 0,19 |
| **Threonine** | 0.682 **±** | 0.165 | 0.617**±** | 0.025 | 2.257 **±** | 0,508 | 0.891 **±** | 0.160 | 0.944**±** | 0.039 | 2.447 **±** | 0,78 |
| **Phenylalanine** | 0.336 **±** | 0.039 | 0.502**±** | 0.005 | 2.234 **±** | 0,619 | 0.424 **±** | 0.112 | 0.675**±** | 0.091 | 1.782 **±** | 0,51 |
| **Aspartate** | 2.097 **±** | 0.639 | 0.831**±** | 0.150 | **72.496 ±** | **18,674** | 1.995 **±** | 0.590 | 1.481**±** | 0.182 | **81.855 ±** | **12,44** |
| **Glutamate** | 3.509 **±** | 1.008 | 3.497**±** | 0.117 | 29.611 **±** | 4,284 | 3.156 **±** | 2.163 | 6.632**±** | 1.153 | 31.542 **±** | 7,27 |
| **Asparagine** | 2.869 **±** | 0.439 | **5.386±** | **0.277** | 14.548 **±** | 0,932 | 7.326 **±** | 3.084 | 17.960**±** | 3.757 | 25.472 **±** | 11,91 |
| **Glutamine** | 5.720 **±** | 0.359 | **3.738±** | **0.081** | 23.379 **±** | 7,211 | 11.463**±** | 1.304 | 13.445**±** | 0.989 | 24.913 **±** | 9,62 |
| **Total** | 19.179**±** | 3.361 | 19.612**±** | 0.003 | **158.810±** | **32,210** | 29.202**±** | 8.863 | 49.120**±** | 4.011 | **181.305±** | **25,61** |

## Results are presented as mean of three biological replicates ± SE. For the statistical tests, the data of turning and red stages were both compared to those of the green stage and were analyzed by one- way ANOVA with Tukey’s posthoc test, p < 0.05. Statistically significant data are highlighted in bold.
