# Supplementary material for: The arbuscular mycorrhizal status has an impact on the transcriptome profile and amino acid composition of tomato fruit
Source: BMC Plant Biol. 2012 Mar 27;12:44. doi: 10.1186/1471-2229-12-44 (PMC3362744; doi:10.1186/1471-2229-12-44)
Supplement: Additional file 6 — qRT-PCR primers list. [file 1471-2229-12-44-S6.DOC]

**Additional file 6**: **qRT-PCR primers list**

| **Gene Name** | **Primer Set** |
| --- | --- |
| **AAT** | AATf CCCCATTTTTGGAGGGATCT |
|  | AATr TTCATGGCCAATGGAGGTAAAC |
| **P1a** | P1af GATCCAGTGGACGGCGATTC |
|  | P1ar AGCAGTACGGCCGAGTCCAG |
| **ALN** | ALNf CCCGTGACATGGACACATGG |
|  | ALNr CCACAGCAATTGCCCCCTTAG |
| **GH3** | GH3f CCTCTCAGCGGCGATTAGCA |
|  | GH3r CTGCAAATCCATCTTCTTCTTTTG |
| **HDC** | HDCf AAGAGGCATGGCACATGTTG |
|  | HDCr GGCAAGGAGGCAGAGCTTTT |
| **TAGL12** | TAGL12f GGAGGCGCAGGAACAATGAC |
|  | TAGL12r TGCAGCTTCCAGTATCCCTTCC |
| **ADC** | ADCf TGCACCACATTCATCCTGCTG |
|  | ADCr TATTGCACATTCTGCGCCAAC |
| **PRS** | PRSf GACGTGCATTCCCGGCTATC |
|  | PRSr GCCAGAGTTCCGGATGTTGC |
| **TPS** | TPSf TGGTCAGTCCGAGCATCATCA |
|  | TPSr TAACCAGCTGGCCGAGCAAT |
| **GPD** | GPDf ATTGGTCCCTGGAAGGACACC |
|  | GPDr GGCATGTGCTCTTGCGATGAG |
| **UGE** | UGEf GACGGACACATTGCTGCCTT |
|  | UGEr CGGCCACCATTTCAAGGACT |
| **GPU** | GPUf CGGCTCAAGTTGGGGAGGTT |
|  | GPUr CCAGCAGAGGCTCCATGGTT |
| **Ssu72** | Ssu72f CCTCATATGGCACTGGGCAAC |
|  | Ssu72r GGCGGAGGTCATCGAACATC |
| **APK** | APKf CAAGCCTGGGACAGGTGTGA |
|  | APKr CTTCTGCCAGCCATTCCCTG |
| **STK** | STKf GCACCTGAAACACCCTGCAAG |
|  | STKf CCGCTTCAGCCCTGATAGGA |
| **Tcp** | tcpf CTCAGAGGCAACAACAACAGG |
|  | tcpr GGTCTTTTACCGGCGGTTT |
| **GOX** | GOXf GCACAAGCGTGCTCATGTCT |
|  | GOXr TTGTTTCTGCTGCCGATGCT |
| **GOX2** | GOX2f CAAGCGTGCTCGGGTTCAAT |
|  | GOX2r TGCCGATGCTGCTCTTGCTA |
| **HSP70** | HSP70f ATTCCCAAGGTGCAGCAGGT |
|  | HSP70r GCAGCACCATAGGCAACAGC |
| **ADF** | ADFf TTCTGGAATGGGTGTGGCTG |
|  | ADFr GTTTCAGCAGGGCTGCCAGT |
